# Zap1 Sticks It to *Candida* Biofilms

**DOI:** 10.1371/journal.pbio.1000117

**Published:** 2009-06-16

**Authors:** Kira Heller

**Affiliations:** Science Writer, Oakland, California, United States of America

Microbes have a variety of strategies to protect themselves from environmental assaults, whether they are clinging to bare rock on windy cliffs or dwelling within multicellular organisms like humans. One such strategy is to join forces and form biofilms, communities of microbial cells and their sticky secreted extracellular matrix that can adhere to a variety of surfaces. This method is used by *Candida albicans*, a fungal pathogen that can cause irritating ailments like yeast infections and thrush, as well as life-threatening diseases in immunocompromised people. *C. albicans* is generally a harmless inhabitant of our mouths, gastrointestinal tracts, and genitourinary tracts, but a spectrum of conditions allows it to invade our systems. Once inside the body, it can grow as a multicellular form, rife with branching filaments (hyphae), that creates biofilms on implanted medical devices, such as intravascular catheters and artificial joints. As it assumes this sessile lifestyle, *C. albicans* also begins to secrete an extracellular matrix that consists mostly of carbohydrate polymers, of which the most common is β-1,3 glucan. *C. albicans* biofilms are often resistant to antifungal drugs and can be the source of serious systemic infections; thus, once an implanted medical device has been colonized by a *C. albicans* biofilm, the device almost always has to be removed.

To learn more about how *C. albicans* biofilms are generated, Clarissa Nobile and her colleagues mutagenized wild-type yeast and then screened for strains with defects in biofilm formation. They then searched for the mutations associated with the defects and eventually identified a *C. albicans* mutant that produced a particularly slimy-looking biofilm. After figuring out that the mutation was in a gene called *ZAP1*, the researchers created a mutant in which the entire *ZAP1* gene was deleted. This deletion mutant exhibited the same slimy biofilm phenotype. To confirm that the slimy phenotype was caused by the loss of Zap1 protein function, Nobile et al. reinserted the wild-type *ZAP1* gene into the genome of the *ZAP1* deletion mutant in a process called gene complementation and found that a normal biofilm appearance was restored.

When the authors took a look at the overall growth and ultrastructure of the biofilms produced by the deletion mutant and the complemented strain, they failed to detect any obvious differences in certain factors, such as biomass or thickness. However, close examination with a confocal microscope revealed that the hyphae of the mutants, in contrast with the straight filamentous appearance observed in wild-type or complemented strain biofilms, often terminated with clumps of unicellular yeast-form cells (compare Figure 2A and 2B in the Research Article). Complementation with wild-type *ZAP1* restored a normal appearance to the hyphae.

Because a slimy, glistening appearance can indicate an overaccumulation of extracellular polymers, Nobile and her colleagues next measured levels of β-1,3 glucan (the polymer most associated with *C. albicans* extracellular matrix in biofilms) produced by the *ZAP1* deletion mutant. They found that the mutant produced 1.5–2-fold more β-1,3 glucan than the complemented or wild-type strains did, indicating that Zap1 inhibits the production of extracellular β-1,3 glucan.

To find out what Zap1 does in vivo, the authors turned to rats that had been implanted with catheters and infected with mutant and wild-type *C. albicans*. They found that the deletion mutant, the complemented strain, and the wild-type strain all produced biofilms in vivo. However, the *ZAP1* deletion mutant produced copious amounts of extracellular material compared with the control strains, including over 3-fold more β-1,3 glucan. The complemented *ZAP1* deletion mutant made substantially less β-1,3 glucan, indicating that Zap1 negatively regulates extracellular matrix production in vivo as well as in vitro.

The authors next turned to microarrays to investigate which genes are regulated by Zap1. They found that genes with lower expression levels in the deletion mutant were involved in zinc homeostasis, adhesion, aldehyde metabolism, and hyphal development. Genes that were more highly expressed in the absence of *ZAP1* were involved in a variety of processes, such as alcohol dehydrogenase activity, carbohydrate transport, cell wall structure, ergosterol biosynthesis, and glucoamylase activity. Further experiments revealed that Zap1 protein binds directly to the regulatory regions of *CSH1* and *IFD6*—genes that express two alcohol dehydrogenases that act as inhibitors of extracellular matrix production. A third alcohol dehydrogenase, Adh5, increased extracellular matrix production. Gca1 and Gca2, glucoamylases that microarray profiling had revealed were more abundantly expressed in the *ZAP1* deletion mutant, were also found to increase the amount of extracellular matrix being produced.

Although Gca1 and Gca2 probably activate matrix production in a relatively straightforward manner by producing β-1,3 glucan fragments from longer polysaccharide chains, the role of the alcohol dehydrogenases is more mysterious. The authors propose that that Gca1 and Gca2 may generate aryl and acyl alcohols (molecules known to play a role in quorum sensing)—a mode of communication used by many unicellular organisms to initiate population-wide responses, such as forming a biofilm.

Zap1 and its target genes constitute a novel regulatory circuit for *C. albicans* biofilm formation. This circuit could be a promising target for strategies to prevent biofilms from forming on implanted medical devices, perhaps with fewer side effects than the often-toxic antifungal drugs that are currently deployed.

**Nobile CJ, Nett JE, Hernday AD, Homann OR, Deneault J-S, et al. (2009) Biofilm Matrix Regulation by *Candida albicans* Zap1. doi:10.1371/journal.pbio.1000133**

**Figure:**
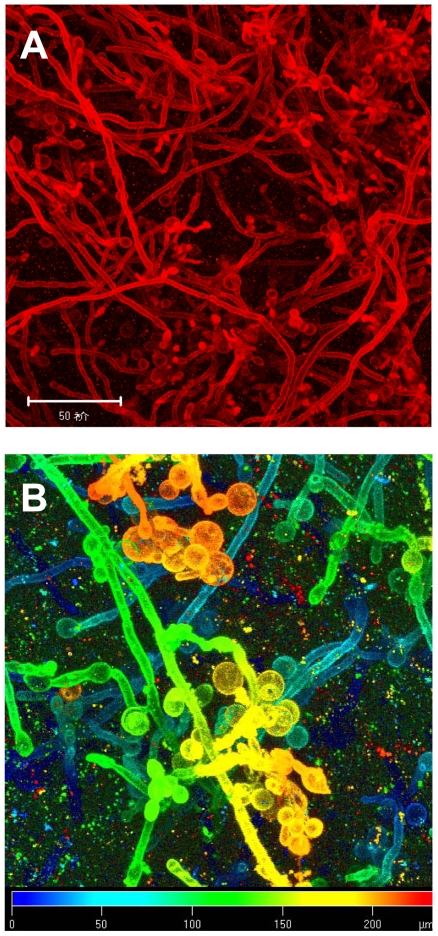
Confocal scanning laser microscopy analysis shows biofilm structures grown in vitro of the *zap1* mutant strain (A) and a projection view with a pseudocolor scale (B).

